# Assessing unstated assumptions of patient and public involvement in palliative care with story completion and typology analysis

**DOI:** 10.1186/s12904-026-02039-7

**Published:** 2026-03-12

**Authors:** Zijian Wang, Michael Chapman, Conal Monaghan, Vinh N. Lu, James O’Connor, Shannon Calvert, Brenda Happell, Imogen Mitchell, Brett Scholz

**Affiliations:** 1 College of Science and Medicine, Australian National University, Canberra, Australia; 2 Canberra Health Services, Canberra, Australia; 3 College of Business and Economics, Australian National University, Canberra, Australia; 4 Perth, Australia; 5 Faculty of Health, Southern Cross University, Lismore, Australia

**Keywords:** Consumer representation, Patient and public involvement, Unstated assumptions, Story completion, Palliative care.

## Abstract

**Background:**

Consumer advocacies emphasize embedding lived experience into palliative care provision. However, tokenistic consumer involvement often hinder meaningful consumer participation in decision-making processes. This study explored stakeholder perception of patient and public involvement in palliative care, identifying barriers and enablers of effective consumer inclusion.

**Methods:**

Using a qualitative story completion approach, we collected narratives written on a fictional consumer engagement scenario by six consumers, seven health professionals, and two palliative care researchers. Analysis revealed underlying assumptions about lived experience contributions and their impact on policy, practice, and quality improvement.

**Result:**

Consumer representatives draw on personal and family healthcare experiences to inform discussions. However, structural barriers and power imbalances often constrain their ability to be meaningfully included. These dynamics contribute to tokenistic representation, particularly within healthcare decision-making committees. Our analysis identified interrelated factors that perpetuate tokenism. By understanding these tokenism-sustaining dynamics of stakeholder interaction, we can develop support mechanisms to shift stakeholder attitudes and foster environments in which consumers are empowered to lead.

**Conclusion:**

To realize the full potential of lived experience in palliative care, healthcare systems must invest in resources and frameworks for targeted training, management structures, and clearly defined roles for stakeholders. Authentic consumer inclusion can transform healthcare systems from tokenistic participation to genuine consumer leadership.

**Supplementary Information:**

The online version contains supplementary material available at 10.1186/s12904-026-02039-7.

## Introduction

Palliative care aims to enhance quality of life for patients and families via a holistic, patient-centered approach [1, 2]. Patient-reported outcome measures and patient preference assessments exemplify the patient centeredness [3]. Individuals with lived experience of illness, along with their family members who provide informal care (carers), are often referred to as “consumers” in this context [4]. Consumers’ treatment journey experience offers critical insights into the effectiveness and appropriateness of care [5]. Such insights are often absent from clinical observations or second-hand reports [6, 7]. These personal experiences can help health professionals understand patients’ journeys, emotions, challenges, and needs from consumers’ perspective [8, 9]. Consumer involvement in palliative care encompasses shared decision-making at the individual level as well as active participation in governance, research, education, and policy [10–13].

Consumer representatives are patients or family carers who participate in formal service planning, governance, or advisory processes with the expectation that they contribute lived-experience perspectives beyond their individual care [9]. There is a trend of involving consumer representatives in the design, delivery, and evaluation of palliative care services with improved collaboration [7, 14–18]. Recognizing consumers’ lived experience has led to meaningful improvements in palliative care [19–22]. Previous research classified three types of consumer consultation: (1) participation – collecting consumer feedback; (2) involvement – recruiting consumers as consultants or research participants; (3) engagement – including consumers in designing and implementing research or services [23–26]. Consumer advocacy further enables consumers to empower themselves, by facilitating consumers to conduct research or manage services as active co-creators, partner and leaders [27, 28].

### Challenges in consumer inclusion

Despite its recognised benefits, consumer involvement in palliative care is frequently constrained by structural, procedural, and relational challenges. These include the lack of clear definitions and implementation guidance [7], limited organisational resources, training, and support [7, 12, 29, 30], and the absence of robust methods to evaluate the outcomes of consumer inclusion [31]. Engagement is further complicated by cultural and ethical considerations across diverse populations [32–35], ongoing difficulties in achieving representative participation [36, 37], and unclear processes for identifying and recruiting suitable consumer representatives [38]. Power imbalances within healthcare systems contribute to tokenistic practices that undermine meaningful involvement [39–43]. These challenges are exacerbated by the emotional and physical limitations of people with serious illness [13, 30, 32, 44–47], the risk of consumer burnout from repeated participation [48], and health professionals’ resistance, role ambiguity, and lack of institutional support for consumer engagement [38, 45, 48, 49].

In addressing these challenges, recent research has clarified definitions of initiatives (structured programs and organisational practices for consumer inclusion) and identified strategies to reduce consumers’ emotional and physical challenges [10, 45]. However, systemic issues like power imbalances and health professionals’ resistance to change remain significant. Addressing these issues creates a paradox. While stakeholders acknowledge improvements are necessary, the entrenched nature of systemic barriers makes meaningful change seem almost impossible without disrupting established roles and structures [50]. Innovating against these intangible challenges requires changing organizational structures, health professionals’ mindsets, and culture, and demonstrated improved outcomes to justify investment [7, 11].

### The role of unstated assumptions

Institutional Theory explains how organizations adopt practices and structures not just for efficiency but also due to external forces (policies, professional standards, community expectations, regulations) [51]. Over time, cultural and cognitive reinforcement embeds these practices in organizations and gradually they are taken for granted [52]. This embedding is not always deliberate or outcome-based; practices may persist because they are habitual or symbolic rather than truly effective. Normalization Process Theory [53] complements this by examining how new practices are collectively enacted and normalized in organizations. Together, these frameworks show healthcare practices and assumptions are shaped by external pressures and internal routines, consciously or unconsciously, normalized.

Many intangible barriers to consumer leadership arise from health professionals’ unstated assumptions about the value and feasibility of consumer input [54]. These assumptions create barriers that are hard to identify and address [55]. In the context of consumer involvement, health professionals’ resistance to changing established practices is a major barrier to the structural changes needed for meaningful consumer participation [56, 57]. Health professionals’ unstated assumptions or implicit attitudes encompass underlying beliefs, biases, or preconceptions that shape their decisions and interactions [58, 59]. Though not necessarily consciously articulated, these attitudes can influence policies [60], care design [61], resource allocation [62, 63], care quality, stakeholder interactions, communication [64], and overall healthcare outcomes [65–68].

Challenges arise when consumer needs and priorities differ from health professionals’ clinical assumptions [69]. Health professionals might undervalue consumer feedback, seeing it as more symbolic rather than substantive [70]. This scepticism leads health professionals to dismiss consumer perspectives instead of including them to enhance improvements [71]. Such assumptions hinder meaningful consumer engagement and create intangible barriers. Health professionals’ resistance may stem from educational gaps, tokenistic experiences, social identity biases, or workload concerns [7, 72–74 ]. Recognising these antecedents is crucial to enact change.

### Current study

Addressing tokenism and promoting collaboration first requires identifying unstated assumptions of stakeholders in committee meeting where decisions on running a hospice were made. This study examined stakeholder perspectives on consumer inclusion in palliative care, identifying key facilitators and barriers using story completion. Specifically, the objectives are:

- To examine stakeholders’ attitudes toward consumer representatives.
- To explore stakeholder understandings of stakeholders’ interactions in palliative care decision-making committee.
- To recommend strategies to enhancing consumer inclusion in palliative care.

## Method

### Design

This study employed the story completion method to examine participants’ perspectives on consumer inclusion in hospice governance [75, 76]. Story completion was used to elicit participants’ assumptions, expectations, and sense-making processes by asking them to complete a partially written hypothetical scenario.

The “committee meeting” referenced in the story stem did not correspond to a real meeting that participants had attended or observed. Rather, it functioned as a narrative device to support participants to reflect on the behaviours, governance processes, and communication practices they might expect to encounter, drawing on their prior experiences. Participants were asked to imagine a decision-making committee through a structured story completion task, rather than to report on a specific event.

Participants completed a story-writing activity in which they were presented with a fictional but contextually plausible scenario involving a multi-stakeholder palliative care committee, comprising care professionals, administrators, and care consumers. Standardised instructions explained that participants would be asked to complete an unfinished story by responding to three open-ended prompts, with no word limit. Each story stem depicted a consumer-related issue arising within a palliative care committee setting.

The story stem described a newly established palliative care committee with consumer representative. Participants were asked to write (1) about consumer representatives’ lead-up to the meeting (2), how other committee members responded in the meeting, and (3) what happened next. This structure allowed participants to elaborate freely on issues of representation, legitimacy, power, and influence.

Story stems were developed collaboratively by a palliative care specialist, a care improvement staff member, and a family carer and were pilot tested prior to data collection. The instructions and story stems are provided in the Supplementary Materials 2. Conventional research methods such as surveys or interviews are often inadequate for detecting resistant attitudes, which tend to be socially undesirable and manifest subtly in health professionals’ behaviours [77–79]. The use of hypothetical context moves participants out of the spotlight, enables participants to project their experience, understandings, assumptions, and expectations into hypothetical situations [80]. This approach facilitates the exploration of beliefs and discourses that may otherwise remain implicit, while also creating a safe and creative space to reflect on potentially sensitive or socially stigmatised issues [81]. Stories are treated not as literal accounts but as meaning-making devices that reflect participants’ underlying cultural and social positioning [75]. The method is particularly valuable as it generates contextual data that can foreground marginalised voices and preserve a diversity of perspectives [82, 83].

Ethical clearance was granted by the Australian National University Human Ethics Team (2019/828). Participants consented to data collection and analysis and were informed of their right to withdraw. No identifiable data were collected. This study is reported in accordance with the Consolidated Criteria for Reporting Qualitative Research checklist [84]. The completed COREQ checklist is provided in Supplementary Material.

### Participants

Recruitment materials were distributed through major national and state-level palliative care organisations and healthcare consumer organizations. Fifteen participants were recruited: 6 carers, 2 palliative care researchers, and 7 health professionals in palliative care. Among the health professionals/researchers, four had < 8 years experience, two had 15–20 years, two had > 20 years, and one did not disclose.

### Data analysis

The analysis was conducted collaboratively by academic and lived experience experts who were carers. One lived experience expert assisted in piloting the story stem, and another contributed to data interpretation and review to ensure that findings reflected consumer perspectives and experiential meanings.

Stories were analyzed using discourse analysis to interpret participants’ epistemologies and unstated assumptions, particularly regarding socially sensitive topics and norms in consumer inclusion, such as assumptions of tokenistic involvement [75, 85]. Discourse encompasses sets of statements and practices that systematically construct subjects and realities within their unique historical and social contexts [86–88]. Discourse analysis examines how language shapes perception, attitudes, and behavior [89]. It also explores the interplay between language, power dynamics, knowledge, social realities, and identity [90–92]. Within this framework, we combined narrative analysis, critical discourse analysis, emotional valence analysis, and typology analysis for a layered interpretation of the data.

All 15 stories were imported into NVivo and supported by supplementary spreadsheets to manage coding. Analysis was conducted collaboratively: a first researcher undertook initial coding and narrative summaries, and a second reviewed, refined, and extended these interpretations. Coding was inductive and iterative, allowing for flexibility in attending to both linguistic and structural nuances.

With narrative analysis, each story was read holistically, with attention to the sequence of events, turning points, and character roles [93–95]. Narratives were interpreted as performances of identity, where participants positioned themselves and others in relation to dominant cultural discourses such as patient autonomy or tokenistic participation [96–98]. This enabled exploration of how individual experiences were embedded in broader social and cultural contexts.

Critical discourse analysis extended the analysis by scrutinizing the linguistic nuances through which power relations and inequalities were reproduced or resisted. Following a socio-cognitive approach [99] and discourse-historical perspective [100], we examined features such as agency, pronoun use, modality, and evaluative language [101–103]. Critical discourse analysis focus on how stories reflected institutional discourses and societal assumptions about palliative care roles, revealing how participants navigated or challenged established hierarchies and social dominance.

Emotional valence analysis was conducted alongside structural and discourse readings, recognising that emotional responses are central to palliative care experiences. Drawing on Cognitive Appraisal Theory, which suggests emotions arises from individuals’ interpretations of events [104], we assessed participants’ event interpretation through the tone and affective content of each narrative. Both explicit emotion words and implicit cues (e.g., metaphors, shifts in tone) were noted, and emotional trajectories were traced across narratives. This enabled us to interpret how participants’ appraisals of situations produced complex blends of hope, fear, frustration, and acceptance [32].

Typology analysis extended beyond individual narratives to identify common patterns of meaning-making and divergent approaches to roles and decision-making [105, 106]. This facilitated examination of similarities within groups and differences between them, providing a higher-level synthesis of the data. To examine whether the typology of story writing differed across participants’ demographic profession, chi-square tests of independence were conducted, which are appropriate for assessing associations between categorical variables.

Together, this multi-layered analytic strategy preserved the richness of individual accounts while situating them within broader cultural, discursive, and emotional contexts.

## Results

We received stories from 15 participants. Story lengths ranged from 81 to 1,071 words (M = 326.6). One participant’s story was excluded due to incompleteness. Three discursive clusters were constructed: representative behaviors, factors influencing efficacy and manifestations of tokenistic representation. Quotations from participants’ stories are labelled by a first digit of the story number/participant code, followed by participant role (consumer [C], health professional [P], or researcher [R]), the temporal stage of the story (before [1], during [2], or after [3] the committee meeting), and, where applicable, a further digit denoting a second [2] or third [3] appearance from the same temporal stage.

### Emerging narratives

#### Representative behaviors: proactively contributing

Participants viewed the representative’s role as a bridge between health professionals and patients, ensuring decisions reflect actual experiences and needs. A health professional participant wrote: “*The members of the meeting were also encouraged to provide an insight into their carer experiences and called upon in the forum to share them* (7P3).” “… *other members acknowledged [the consumer’s] expertise through lived experience but noted that everyone’s experience is unique…* (6R2).” In these extracts, consumer representatives played a crucial role in healthcare management by sharing personal experience to shape discussions and highlight systemic challenges.

Stories depicted consumers representatives identifying problems and proposing solutions. They prepared for meetings by consulting other consumers for diverse perspectives and seeking health professional advice: *“[The representative] sat down to write his thoughts out because he knew that he really needed to be clear in the next meeting*,* worried that most of the committee members wouldn’t get it if he wasn’t measured and considered and careful with his wording”* (2P1). Stories depicted consumer representatives with high aspirations, actively improve services, propose initiatives, and share personal experiences.

#### Influences on the efficacy of representatives

Three factors influenced a consumer representative’s effectiveness: consumer characteristics, external barriers/facilitators, and organizational power dynamics. The following section explores how these factors shaped consumers’ capacity to contribute.

### Consumers characteristics

Four factors affected representatives’ effectiveness: peer support, motivation, emotions, and representativeness.

#### Peer support among consumers

Consumer representatives derived substantial support through interactions with other consumers and carers. These interactions provided crucial emotional and practical support: “*[The consumer representative] called [other] healthcare consumers and had a meeting with them. They were very supportive and listened intently to what [consumer representatives] had to say* (10C3).” Shared experiences fostered a network that helped representatives manage personal loss and the emotional challenges of their roles. “*What would be the best way for me to deal with this*,* I can’t just let it go*…*I’ll talk to [fellow consumers]*,* find what their experience was…at least then I will be talking about more than just my story*…(5P1)”. Such support fostered emotional resilience and engagement, enhancing their participation.

Consumer representatives with a strong sense of belonging contributed more effectively. The establishment of consumer representative subgroups emerged as a particularly successful strategy: “*The Palliative Care Committee developed a sub-group of consumer representatives*,* which met bi-monthly*,* and from this sub-group*,* a patient and carer representative were members of the Care Committee… It was a huge success* (4C3).” These subgroups provided valuable opportunities for representatives to consolidate their perspectives before presenting them to the broader committee.

“*I was a bit nervous that [health professionals] wouldn’t let [the other two consumer representatives] in…. thank God they did. At least now they can see that I am not alone in this. More representatives from carers at the next meeting they suggested - a good idea “cause it is hard to speak my mind in front of all of those educated people*,* better to have a group of us* (6R22).” This explained how having multiple representatives can change communication dynamics with professionals.

In stories 5P1 and 6R22, peer support boosted representatives’ confidence and comfort speaking up. Increasing the number of consumer representatives in meetings eased individual anxiety and brought in more diverse consumer perspectives, strengthening the collective voice.

#### Consumers’ intrinsic motivation to contribute

Stories frequently portrayed consumer representatives as highly motivated and deeply committed, driven by altruistic intentions for systemic change:

“*[The consumer representative] was composed and unwavering in his belief. He did not pass his power back to the hierarchy to approve or disapprove of him or his perspectives*,* but rather took the approach of helping all the future carers that would travel the path he had just been on* (11C2).”

In Extract 11C2 and four other similar narratives, representatives were motivated by an altruistic desire to improve care for future patients and families. Their motivation stemmed from personal experiences and profound empathy, transforming personal grief into advocacy for change:

Personal loss emerged as a profound motivation, with committee participation serving both as a tribute to lost loved ones and a source of renewed purpose: “*I’m doing this for you*,* [Care recipient]. Gives me a reason to stay here*,* a purpose. I still miss you*,* [Patient]*,* [it] just doesn’t hurt so much when I feel I can do something to help another family… I love you*,* my son* (5P3).”

#### Emotional landscapes

A discursive repertoire mentioned that representatives face emotional challenges that affect their ability to contribute. We distinguished a range of negative emotions including nervousness, uncertainty, worry, distress, frustration, and a feeling of disconnection.

In committee settings, representatives frequently experienced heightened anxiety when judged by powerful health professionals with strong viewpoints: " *When [the consumer] stopped speaking*, *there was silence in the room. [The consumer] felt his heart racing and he wanted to run away* (9C2). *"* This nervousness arose from fears of being seen as a token gesture, leading to cautiousness.

The emotional intensity was evident, with characters described as *“crushed* (9C32),” “*demoralised* (9C33),” and *“disheartened* (11C1),” after negative feedback. Extract 9C2 along with similar incident in story 2P noted physiological responses of a “*pounding heart*” (2P23) when speaking. Four stories described the personal grief faced by family carers from different perspectives, for example: *“Others raised concerns regarding the consumer*,* would this impact on their mental health and grieving process*,* stating it might be upsetting for the individuals* (4C2).*”* One of the carer participants wrote that representative *“put on armour* (9C1)*”* to attend meetings in their grieving.

Positive emotions like empowerment and resilience were described in eight instances: *“[The consumer] felt calm and confident*,* and very connected… He practiced silent deep breathing as the meeting was formally opened… he felt relief* (11C23).” These descriptions of commitment and confidence depicted representatives deeply driven to have their stories heard and to make an impact (seen in stories 5P, 11 C, and 13P).

#### Representativeness of consumers

Stories debated whether a single consumer can adequately represent the broader consumer voice. Stories emphasized issues of diversity: individual insights are valuable, but a range of lived experience is necessary. Three stories covered gender and racial minority representation, noting how these groups are underrepresented: “*[The consumer] felt frustrated and disheartened by the committee’s response to his involvement… was it because caring was typically associated with a female role and as a male his caring abilities were somehow invalidated?*,* Or was it because his partner who had he lost was male*,* making it somehow difficult to relate to other caring situations at the hospital?* (11C12).”

Similar narratives underscored that most carers are women, and they questioned whether male carers (husbands, same-sex partners) or carers from minority groups could represent all carers’ experiences. Still, story 2P and 11 C argued that minority representation is necessary and that a diverse committee helps comprehensive understanding. Notably, these doubts came from the representatives themselves rather than from health professionals in the narratives. Many consumer representatives actively gathered input from diverse consumer groups to present multifaceted views with collected efforts, as previously discussed in 2P, 5P and as in 10C3.

A health professional participant questioned the notion of comprehensive representation in their story: *“Did Dr. Andrews - the chair of the committee - represent all physicians? Did the policy officer from the health board represent all policy officers? Why was everyone so keen that [a consumer] ‘represent’?!* (2P12)*”.* This critique challenged the expectation that one consumer should speak for all patients and carers. Ultimately, these narratives underscored skepticism about the validity of representativeness of a single consumer.

### External barriers

#### Resource constraints

Stories highlighted three types of resource constraints. First, representatives struggled with both tangible and intangible resource deficiencies in their representative roles: *“[The consumer] had gotten the impression that they wanted him to do the impossible - represent consumers in general even though they weren’t giving him resources to go and seek other consumers’ perspectives* (2P13).” Without support, consumer representatives often had to prioritize other life commitments, reducing their impact.

Second, organizations struggled to implement consumer recommendations due to resource shortages. Many agreed-upon meeting outcomes were never implemented: “*… the initiative wasn’t given resources to be developed*,* and never took place* (8R3).”

Third, there was a lack of investment in consumer representative human resources. In particular, training shortfalls emerged as a key barrier to consumer representatives’ preparedness. This shortfall was either self-acknowledged by the representatives themselves, *“He didn’t know how to explain his thoughts about needing to be “representative” in a meeting full of people with very strong views about what kinds of representation were needed* (2P14)*”*, or evident in their uncertainty about their role, indecisiveness, lack of professionalism, and limited understanding of the palliative care system: *“[The consumer] wanted to make sure she had an understanding of the different perspectives already represented at the meeting and what they thought about carer representation at this meeting* (7C1)*”*. Such descriptions underscored the lack of comprehensive orientation, training curriculum, or supervision. Eight stories indicated a perceived systemic under-resourcing for all three aspects.

#### Health professionals’ role ambiguity

Narratives highlighted health professionals were reluctant to take on extra responsibilities and were unsure of their roles in consumer inclusion. They were concerned about the workload of engaging with consumer representatives and implementing outcomes.

Recurring narratives indicated that health professionals’ additional workload for managing consumer inclusion was not acknowledged, “*the majority [of health professionals] had concerns about how this level of engagement could be managed and who would be responsible* (1C2).”, and health professionals saw it as an untenable additional workload: “*[The health professionals] who [consumers] know to mean well but get easily sidetracked by [health professionals’] multiple responsibilities and competing priorities* (13P1).”

Unclear role definitions contributed to these challenges. No story described any processes, dedicated time or designate responsibilities for integrating consumer inclusion. In 1 C and 10 C, the lack of clear roles and organizational support linked with health professionals’ reluctance or inability to involve consumers effectively.

#### Institutional systems and processes

Structural and institutional factors also shaped consumers’ contribution in the stories, including management procedures and communication protocols. One health professional participant wrote about the impact of inadequate meeting documentation: “*While [consumer] thought this was a good idea*,* the discussion seemed to get derailed and there wasn’t any sort of resolution (2P2).”* Without proper documentation, discussions became unfocused. Another story noted that an overly rigid agenda limited the representative’s participation. Two participants’ characters indicated preference for more flexible engagement methods, such as carer focus group (7P) and interview (13P).

Well-structured consumer involvement was perceived as capable of influencing practice change, underscoring the importance of strong institutional support:

*"The [Palliative] Care Committee developed a sub-group of consumer representatives*,* which met bi-monthly*,* and from this sub-group a patient and carer representative were members of the [Palliative] Care Committee. The End-of-life Care Committee reviewed guidelines and changed practice (if needed) to meet consumer participation. The intent of the End-of-life Care Committee was clarified*,* and is a high-level meeting to consider regional approaches to end of life care. It was a huge success* (3P3)."

### Power dynamics

#### Hierarchies, exclusionary practices, and disempowered consumers

The way of organizational exclusionary practices presents challenges for representatives. In four instances, stories covered strong health professional solidarity particularly among physicians. For example, a carer participant wrote: *“[The consumer representative] suspected that the doctors stuck together and*,* therefore*,* were not going to criticise each other*,* especially in public (9C2).”* This physician solidarity was seen as creating an environment unwelcoming to critical discussion or challenges to established practice.

Perceived disparities in knowledge and status reinforced a sense of alienation in meetings: “*[The consumer] pondered about the committee’s reluctance over his involvement. Was it because he was not a recognised … professional or academic and therefore had no right to have his voice heard?* (11C13)”. This alienation was linked to negative emotions like anxiety and intimidation (see 11C1, 9C2, 2P23).

During the meeting, consumers remained peripheral, with minimal influence. They were portraited as lower in the hierarchy than other committee members. Representatives found it difficult to have an opportunity to speak: “*[The consumer] put his ha*n*d up to speak as he didn’t feel like he would get a word in otherwise* (9C23).” Twelve stories depicted a committee dominated by executives. A carer participant wrote: “*decisions were made on behalf of consumers rather than with consumers* (14C1)*”*. At least seven stories narrated a lack of genuine power-sharing.

After meetings, consumers had little power or control over outcomes. In four stories, consumer representatives couldn’t get their suggestions implemented. When change did occur, they were usually driven by other powerful figures:

*“The following year*,* when [a health professional] as chair ended*,* [a new doctor] was elected as the incoming chair. [The new doctor] had occasionally wondered about the discussion about consumer representation on the committee*,* and endeavoured to discuss it in her first meeting as chair. She raised the issue of representation and the new committee seemed receptive to the idea of having more representatives* (2P3).*”*

In Extract 2P3, the new committee showed more openness to expanding representation. This change was driven not by consumer advocates themselves, but rather by a pro-consumer health professional who took on a leadership role within the committee. Only two stories narrated consumer representatives themselves successfully persuading other stakeholders.

#### Care professionals’ attitudes and actions

Almost all stories explicitly or implicitly depicted health professionals as entitled, superior, and inflexible in their perspectives. Story 2P, described doctors citing the past absence of consumer representatives as proof that such consumer inclusion was unnecessary. This defensive posture suggested resistance to consumer representation, seeing it as an unwelcome change to established practices.

Resistance to new perspectives were also evident through non-verbal cues and behaviors. Dismissive behaviors like negative body language reinforce these perceptions of health professionals’ disengagement: “*The meeting was a disaster… All the big wigs arrived either just on time or late*,* wearing suits*,* parading like peacocks!!* (9C22)”. This depicted an imbalanced power dynamic, casting health professionals as uncooperative and dismissive.

Two narratives mentioned health professionals resisted consumer involvement due to an education gap between consumers and health professionals. For example, in Extract 11C13, consumer’s lack of medical training led health professionals to dismiss his input, causing self-doubt and withdrawal from the committee. This was evident when health professionals questioned the feasibility of laypersons’ proposals, revealing a bias against perspectives outside the medical framework.

However, some narratives involved health professionals’ support and acceptance. Five stories (e.g., 3P2 and 3P3) described gradual positive shifts in health professional attitudes. Health professionals began acknowledging issues, reassessing structures, considering alternative views, and addressing consumer representatives’ concerns: *“[Consumers’] concerns were listened to and were supported… Consideration of a separate committee was discussed…* (3P2)”. Health professionals also explicitly appreciated consumers’ efforts (as in 6R2), indicating growing receptivity. These narratives highlighted an openness to expanding consumer representation, suggesting optimism among participants.

Health professional attitudes were often the main criterion for judging consumer inclusion in five stories. For example, when a participant wanted to wrap up their story with an optimistic ending: *“[The representative] hoping it would be more successful now that more people would listen to her* (14C31).” Or as in another story *“This [management innovation] was achieved successfully and all committee members were happy* (12P3)*”.* Story 14 C by carer and 12P by health professional, treated health professionals’ approval as the measure of consumer inclusion success. These narratives suggest participants believed that health professionals’ acceptance or resistance ultimately determined the value of consumer participation, despite consumer centeredness in palliative care.

#### Policy and government stakeholders

Policy and government figures appeared in five stories, with three involved committees engaging with healthcare policymakers and government regulations. The following extract and similar narratives involved participants’ perception of policy experts as arbitrators when facing health professional resistance:

“*[The consumer representatives] organised a meeting with the Minister for Health and explained the situation. The Health Minister was concerned about the culture around consumer representation not being valued and that people were dying badly… [The Minister] also met with the end-of-life care committee and kicked ass and said that [the consumer] needed to be listened to and that his input was the most important for this group* (9C3).”

In Extract 2P3, the powerful figures Health Minister used their authority to ensure the consumer was heard. This suggests participants saw policymakers at the top of the hierarchy and tried to leverage these authorities to push for consumer input when health professionals resisted.

Other stories showed consumer representatives proactively engaging with policymakers to bolster their influence in the committee. “*[The consumer] spoke of the types of policies and procedures that would need to be revisited to allow better integration of carers and patient input and how this was essential for the person dying getting the end of life they desired* (11C22).” Across these narratives, participants often had a simplified view of government and policymaker: they saw policymakers as authority figures who could directly control healthcare practitioners, not fully recognizing the complex stakeholder and regulatory landscape.

### Tokenistic representation

Our analysis identified four common pathways that perpetuate tokenistic consumer involvement. This section examined four common sequences of events that collectively perpetuate superficial consumer inclusion.

**Fig. 1:**
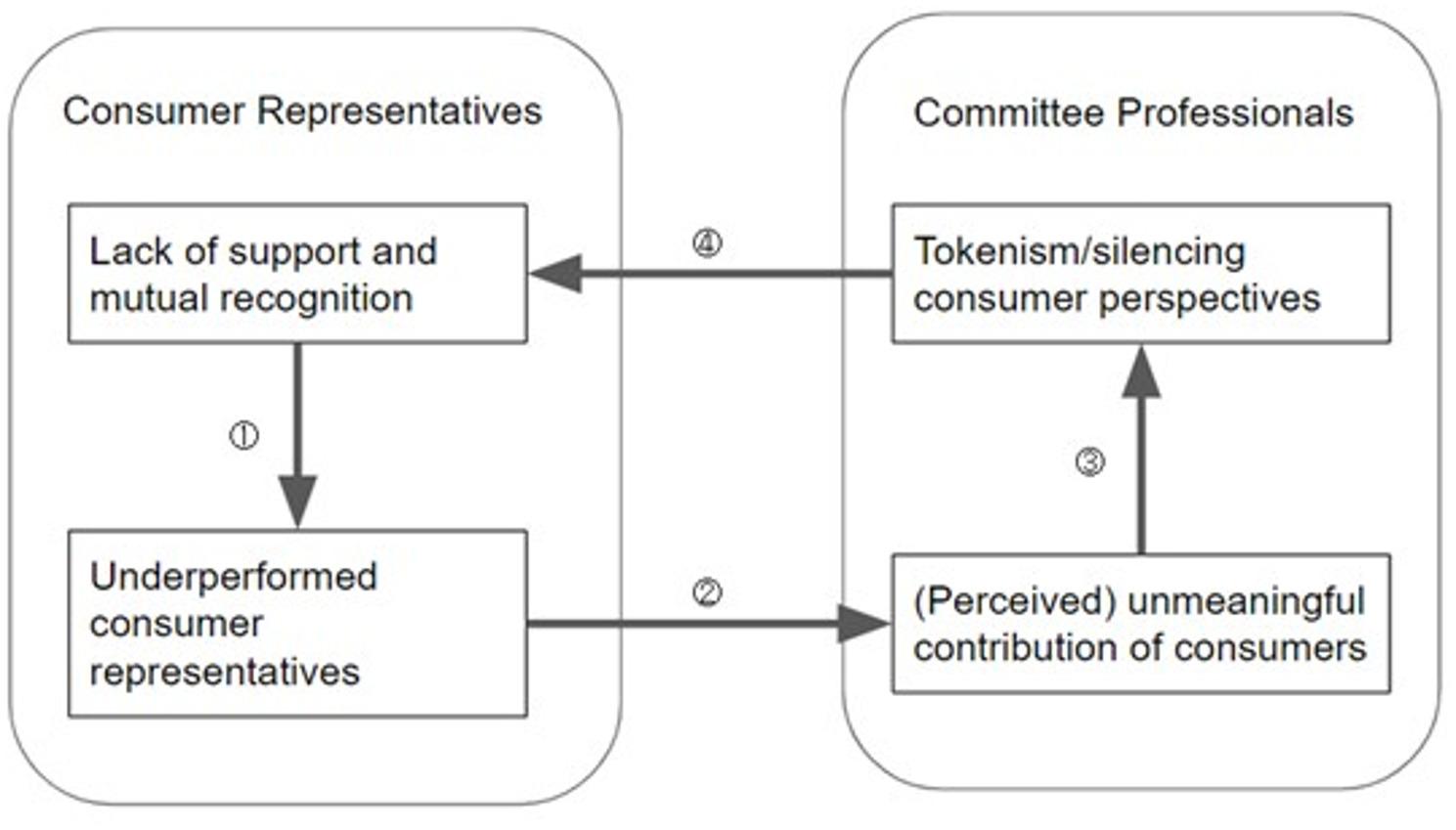
Dynamics of consumer participation and cokenism

Each of the following four sections corresponded to one of the four pathways illustrated in Fig. 1, as indicated by the matching circled numbers that appear both at the beginning of each paragraph and on the respective pathway in the figure.

#### ① Representatives’ challenges stemming from inadequate support

Stories narrated consumer representatives faced major challenges: lack of financial resources, limited engagement opportunities, and poor access to information. Extract 2P13 described financial challenges hurt consumers’ preparedness and attendance. Some narratives described representatives experiencing anxiety and questioning their legitimacy in their roles. *“[The consumer] doesn’t feel empowered to prepare for [the representative role] at all…* (14C12)” highlighting systemic barriers that hinder preparation and make it difficult for consumers to navigate complex systems.

Even if a consumer representative had an isolated success, without systematic support and backing, their overall effectiveness was compromised: *“[The consumer] had limited resources and limited support but was able to convince someone in the executive …* (10C32)*”*. In stories 9 C and 11 C, lack of training left consumer representatives struggling to navigate the complex healthcare system. Other narratives (e.g., 14C12 and 10C32) suggested consumer representatives often felt unprepared to convey their perspective in a meaningful way.

#### ② Misalignment between consumers’ contributions and care professionals’ objectives

Stories often portrayed that consumers, lacking understanding of care intricacies, proposed solutions outside the committee’s scope: *“some [health professionals] have been on a committee where the consumer rep is one of the dominant personalities who take up time in the meeting but don’t help find actionable solutions* (13P2).*”* This suggests a disconnect between health professionals’ expectations and the value of consumer inclusion. Health professionals primarily value solutions within current constraints, whereas consumers provide lived-experience perspectives that challenge existing frameworks and highlight unmet needs. What seems like *“taking up time”* without *“actionable solutions”* reflects the tension between immediate practicality and the need for fundamental system change.

This misalignment frustrated all stakeholders and diminished the perceived value of consumer inclusion. “*Some [health professionals] are really welcoming of the representatives but at a loss as to how to engage them when the issues they raise are outside the control of the committee* (13P22).*”* This highlights how knowledge gap contributed to disconnects between consumer priorities and what the committee could realistically address or influence. In stories such as 2P, 5P, and 7P, consumer inclusion seemed ineffective not due to lack of insight, but because of healthcare system barriers. These barriers included rigid committee processes designed mainly for clinical input, insufficient resources to act on consumer suggestions, and institutional resistance to perspectives challenging status quo. Exacerbating this challenge, insufficient training and the knowledge gap between consumer representatives and health professionals further diminished the perceived value of consumer input, as seen in story 11 C.

#### ③ Perception of tokenistic engagement and limited impact of consumer participation

The procedural nature of committee meetings may limit the potential for consumer inclusion to influence outcomes: *“Meetings often start with high hopes but result in the same list of risks and objectives*,* leading to a perception that discussions are cyclical and do not effectively address or resolve critical issues* (13P23).*”* This suggests that no matter how well-intentioned, consumer input will have little impact unless the committee’s own processes can produce meaningful change. Challenges with consumer inclusion were frequently noted, yet explicit health professional recognition of its value appeared only in Story 6R. This might indicate a systemic tendency to undervalue consumer inclusion.

A narrative by a health professional illustrated how consumer inclusion can be perceived as tokenistic when they are not meaningfully integrated into committee processes: *“… the discussion seemed to get derailed and there wasn’t really any sort of resolution… [representative] stayed on the committee for a few months but didn’t feel like he was really valued*,* so eventually excused himself* (2P22).*”* The representative’s withdrawal highlights that feeling devalued can silence consumer voices, reinforcing a cycle where participation exists only in form. Such experiences reinforce perceptions of tokenism. These narratives imply a cycle: when health professionals undervalue input, consumers disengage, which then reinforces health professionals’ view that consumer participation has little value. Perceiving consumer input as having little impact further reinforced the view that it was merely tokenistic.

#### ④ Recognition and support: impact on consumer representative engagement and effectiveness

Lack of genuine recognition and support made consumer representatives doubt the value of their contribution: *“Execs make decisions on behalf of consumers… [the consumer representative] wondered whether to bring it up… but knew it would be pointless* (14C2).*”* This quote illustrates how a perceived lack of decision-making power can lead consumer representatives to self-censor, ultimately reducing their role’s significance within the committee. “*The majority [health professionals] had concerns about how this level of engagement could be managed and who would be responsible* (1C2).*”* These quotes suggest that if health professionals see little value in consumer inclusion, it becomes a self-fulfilling prophecy: reduced consumer effectiveness then reinforces the health professionals’ skepticism.

Together, these four pathways suggest a self-reinforcing cycle. The narratives indicate that poor recognition and support undermine consumer effectiveness, which then seems to validate low perceptions of consumer inclusion value, further eroding support. These story completions suggested feedback loops that contribute to superficial consumer inclusion. Nevertheless, as a story completion study, this reveals participants’ perceptions of these dynamics but does not prove causality.

### Typologies of consumer contributors and associated narratives

We constructed two sets of typologies from the narratives: styles of representative contributions and narrative construction patterns.

#### Typologies of representative actions

We identified three distinct contribution style, each with different approaches and outcomes (Table 1). These typologies reflected the different ideologies participants expressed in their stories. Chi-square test showed no significant link between contribution style and participant type (carer, health professional, researcher), χ²(4) = 5.60, *p* = .23.

**Table 1:** Contribution styles of main character

Stakeholder Type	Evidence-based Contributors	Emotional and Experiential Contributors	Systemic Change Advocates
Characteristics	Recognizing the value of diverse perspectives to generate best quality contribution; some demonstrate strong emphasis on the use of data and research	Leveraging lived experience; emphasizing personal emotional and experiential insights	Focus on addressing systemic barriers and executive-level resistance; demonstrating strategic thinking in overcoming institutional challenges
Primary Actions	Conducting thorough meeting preparation; presenting evidence-based arguments; integrating research findings	Sharing personal experiences of palliative care	Developing innovative engagement methods; proposed alternative participation mechanisms (e.g., question and answer sessions, remote participation options)
Contribution Impact	Influencing palliative care policy and practice through comprehensive consultation processes and evidence-based communications	Highlighting human-centered aspects of palliative care; shaping policy and practice through authentic lived-experience perspectives	Enhancing consumer empowerment in organizational decision-making processes; challenged existing power structures

#### Typologies of stories

**Fig. 2:**
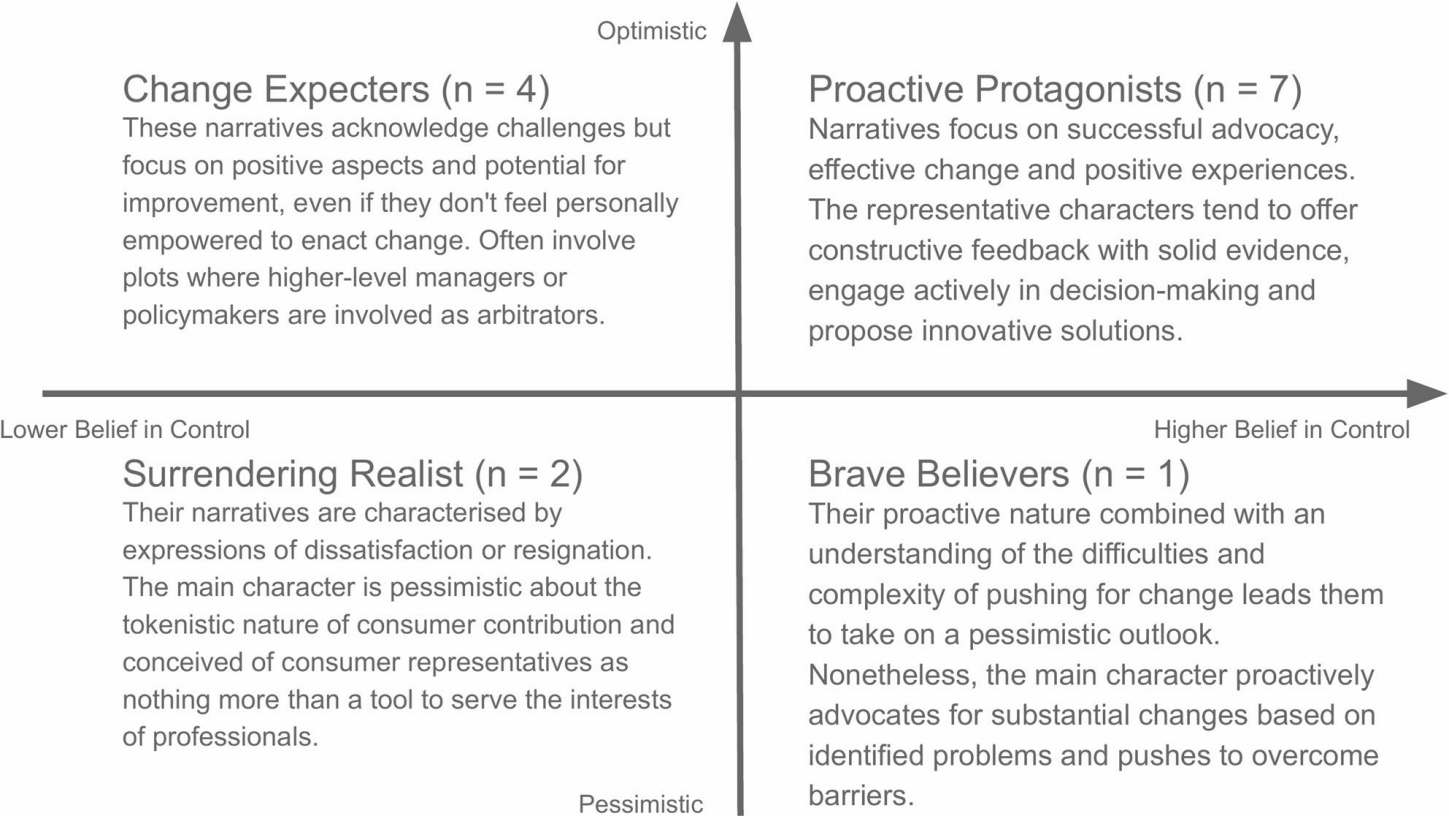
Typology of narratives

The analysis categorized the stories along two dimensions: the level of consumer representatives’ perceived over committee outcomes (high vs. low) and the type of story ending (optimistic vs. pessimistic). These dimensions formed a matrix for narrative pattern classification (Fig. 2]. The distribution of narratives reflected participants’ nuanced understanding, recognizing both the potential and challenges of consumer involvement.

Most stories ended on an optimistic note (11 of 14), though three stories ended pessimistically with unresolved issues. The chi-square tests showed no significant associations between locus of control (internal vs. external) and participant demographic, χ²(2) = 0.39, *p* = .82, nor between ending type (optimistic vs. pessimistic) and participant demographic, χ²(2) = 1.13, *p* = .57.

## Discussion

### Overview of findings

Most narratives showed consumers progressing from initial apprehension to finding their voice in decision-making committees. Although consumers are motivated by altruism, empathy, and a commitment to future patients [107] (see 11C2), they are frequently denied opportunities for meaningful participation [108, 109]. In our interpretation, consumers’ proactive efforts were often sidelined by dominant health professional voices. Representatives faced challenges like anxiety, uncertainty, and grief. These struggles were often worsened by practical issues, including inadequate training and insufficient resources.

Our findings show that health professionals’ sense of entitlement and clinical focus can create barriers to meaningful consumer inclusion. In many narratives (e.g., 9 C and 11 C), health professionals indirectly undervalued consumer inclusion. The challenge of hierarchy of power [110] was evident in our “Hierarchies” narratives. Notably, participants often viewed policymakers as allies against resistant health professionals (e.g., Extract 9C3). This suggests external pressure can help overcome entrenched hierarchies that consumer representatives face from within.

We also identified distinct ways participants conceptualize consumer inclusion [106]. Our refined three-role typology (evidence-based contributors, emotional and experiential contributor, and systemic change advocates) distils a previous five-role model: partner, expert, advisor, advocate, and personal engagement [111] into a streamlined framework, capturing the essential commonalities while remaining flexible for different contexts and levels of consumer empowerment.

While this diversity of roles provides many perspectives, it can misalign with committee expectations [112], as shown in our “Misalignment” pathway. For example, extract 13P2 described a health professional seeing a consumer rep as a “*dominant”* figure who *“takes up time. but [doesn’t] help find actionable solutions*,*”* which highlights the disconnect between immediate health professional priorities and the consumer’s systemic focus [113]. As illustrated in stories 3P and 6R, addressing this misalignment requires two strategies. First, committees should adopt more flexible structures (e.g., the consumer subgroups in extract 4C3). Second, consumers need training and support to navigate health professional environments [114], which would close the gaps noted under “Resource Constraints”.

Our narrative typology (Fig. 2), which classified stories by perceived control and ending tone, showed that 11 of 14 stories were optimistic (“*Proactive Protagonists*” or “*Change Expectors*”), reflecting confidence in the value of consumer input despite systemic barriers. Yet the “*Resigned Realists*” group and tokenism pathways (Fig. 1) revealed lingering doubt that such input would be acted upon. The narrative typology pattern parallels earlier analyses of stakeholder discourse on lived-experience contribution, which identified four positions: *patients as experts*, *skills and representation*, *self-protection*, and *professionals know best* [115]. Our two-by-two matrix refines these categories by integrating both consumer and professional perspectives within a clearer articulation of the antecedents and theoretical positioning of the discourse.

Carers, professionals, and researchers appeared to draw on overlapping discursive resources when constructing their stories, indicating shared interpretive repertoires around consumer participation. This aligns with the view that meaning is co-constructed across social positions [116, 117]. While no distinct role-based patterns were evident in this analysis, this does not suggest that participants’ roles had no influence. The analysis was undertaken holistically, treating all stories as an integrated interpretive field rather than as discrete groups, consistent with holistic approaches to narrative inquiry [118].

### Theoretical contribution

This study extended Institutional Theory [51] and Normalization Process Theory [53] by showing how implicit health professional attitudes become embedded in healthcare routines and perpetuate power imbalances. We observed that health professionals’ resistance to change is often subtle, for example, dismissive body language or disengagement (Extract 9 C), and such behaviors help maintain current hierarchy.

Using story completion allowed us to uncover “unarticulated assumptions” [60] that traditional methods might overlook. We found that disparities in education (Extract 11 C), tokenistic representation (Extract 2P), and workload pressures (Extract 13P) can constrain meaningful consumer inclusion, supporting previous findings [7, 73, 119] while adding contextual nuance. However, story completion does not aim to capture individual lived experiences of consumer involvement [120]. Accordingly, we do not suggest that these narratives mirror the realities of consumer participation or provide authentic or verifiable accounts of consumer–professional dynamics in leadership settings.

Tokenism remains a major concern in patient/public involvement [121, 122]. Our narrative-based framework (Fig. 1) illustrates intangible barriers to consumer participation, mapping four linked pathways that marginalize consumer representatives. It suggests that tokenistic collaboration can be both a cause and an effect of consumers being perceived as ineffective. As the “Collaborative Strength” narratives showed, breaking this tokenism cycle requires robust supports (training, peer networks) to empower consumers. In turn, empowered consumers can help foster organizational attitudes that genuinely value lived experience.

There have been substantial debates about the representativeness of consumer representation. Many argue that consumers’ value lies in connecting to the broader community, rather than being a sole spokesperson [38, 123, 124]. Our findings echoed these debates, while showing representing is not feasible for most consumers (see extracts 2P, 10 C, 11 C). Moving forward, clearer expectations and perhaps reframing the role from “representative” to “leader”, “consultant”, or “ambassador” could help.

### Practical implications

#### Improve training for consumer representatives

Our analysis found consumer representatives contributions often misalign with health professional committee expectations, indicating a need for training to improve system knowledge, technical skills, and emotional preparedness. The lack of structured support we observed [125] left consumer representatives feeling unprepared and unsure of system processes. Better training would also help consumer representatives manage the emotional challenges of the role (e.g., coping with grief as in stories 9 C and 11 C) and translate their lived experience into constructive solutions.

#### Establish frameworks to guide health professionals

We observed that without clear processes, health professionals can feel overburdened or unsure about engaging consumers (see extract 1C2). Organizations need to implement training and support frameworks for staff [126] that clarify who is responsible at each stage of consumer engagement (selection, consultation, implementation, evaluation). Importantly, health professionals should be given dedicated time and support to incorporate consumer input, rather than simply adding new duties to an already full workload.

Tokenistic practices often start at the leadership level [127], and our findings concur. For example, in extract 14C1, a lack of senior management support led to tokenistic plans and misallocated resources. Meanwhile, frontline staff who do value consumer input [11] often lack the authority to change how resources are used. It’s crucial that top decision-makers understand the tangible benefits of genuine consumer involvement. This will help ensure proper resources and support are allocated, reducing tokenism.

#### Diversify engagement formats

Relying solely on committee meetings can reinforce hierarchies and intimidate consumers, as we saw with the anxiety responses in extracts 9C2 and 2P23). Offering more flexible, consumer-friendly formats can empower participation [114]. In our study and previous literature, people suggested alternatives like one-on-one interviews, Q&A forums, written feedback, exit interviews, workshops, co-creation sessions, and focus groups (Extracts 7P, 13P). Such formats create less formal, more egalitarian environments [128] and give consumers greater leadership [129, 130]. Practical steps include running dedicated workshops or focus groups, forming consumer advisory committees, or setting up one-on-one consultation sessions [131–135]. These approaches answer the call for less formality, less hierarchy, and stronger consumer voice.

Relatedly, expanding engagement beyond traditional committees can also help address resource limitations. Authentic consumer leadership is crucial to avoid tokenism but requires further commitment [54, 71]. Using alternative models such as smaller group approach (Extract 3P3) can allow more inclusive, cost-effective consumer involvement that is tailored to participants’ needs.

Lastly, our use of discourse analysis helped uncover implicit assumptions that would otherwise remain hidden. These were not explicitly stated by participants but came through in how they told their stories. For instance, using deferential language toward health professionals, downplaying consumers’ agency, or showing frustration when ignored, all of which hint at underlying beliefs about authority and legitimacy in the process. In short, many of the attitudes were implied by narrative tone and framing rather than openly declared.

We also note that variation in storytelling skill or length did not diminish the insights provided. Both concise and detailed narratives offered valuable contributions, addressing concerns that variation in story length might constitute a methodological limitation [76, 136]. Our sample size was small and all 15 participants were in Australia, therefore these findings reflect palliative care context of a western developed country. Its transferability to other countries requires cautious.

## Conclusion

This study outlined key challenges and opportunities for enhancing consumer inclusion in palliative care. Consumer representatives often remained marginalized, even as they moved from initial nervousness to finding their voice within healthcare hierarchies.

Using story completion alongside discourse analysis allows detections of implicit assumptions and power dynamics that traditional methods might miss. Tokenistic inclusion has multiple underlying causes and breaking the cycle of tokenism requires stronger structural support to truly empower consumers.

Our findings suggest practical ways to integrate consumer perspectives more authentically into healthcare, making systems more equitable and responsive to lived experience. By shedding light on both the obstacles and the opportunities, this study lays a foundation for transforming palliative care into a more inclusive, responsive field, one where lived experience is genuinely valued and drives real change.

## Supplementary Information

Supplementary Material 1. COREQ (COnsolidated criteria for REporting Qualitative research) Checklist

## Data Availability

All data are available from the corresponding author upon reasonable request. This includes original data, data extraction forms, and analysis datasets. Study materials are available in supplementary materials.
