# Supplementary material for: Assessing unstated assumptions of patient and public involvement in palliative care with story completion and typology analysis
Source: BMC Palliat Care. 2026 Mar 12;25:115. doi: 10.1186/s12904-026-02039-7 (PMC13112636; doi:10.1186/s12904-026-02039-7)
Supplement: Supplementary file 1 — Supplementary Material 1. COREQ (COnsolidated criteria for REporting Qualitative research) Checklist [file 12904_2026_2039_MOESM1_ESM.docx]

**Supplementary Materials**

## **Supplementary Material 1: COREQ (COnsolidated criteria for REporting Qualitative research) Checklist**

Consolidated criteria for reporting qualitative studies (COREQ): 32-item checklist

Developed from: Tong A, Sainsbury P, Craig J. Consolidated criteria for reporting qualitative research (COREQ): a 32-item checklist for interviews and focus groups. International Journal for Quality in Health Care. 2007. Volume 19, Number 6: pp. 349 – 357

| **Item No** | | **Guide Questions/Description** | **Reported on Page #** |  |
| --- | --- | --- | --- | --- |
| **Domain 1: Research team and reflexivity** | | | |  |
| **Personal Characteristics** | | | |  |
| 1. Interviewer/ facilitator | | Which author/s conducted the interview or focus group? | N/A for the story completion task is survey based |  |
| 2. Credentials | | What were the researcher’s credentials? E.g., PhD, MD | N/A for the story completion task is survey based |  |
| 3. Occupation | | What was their occupation at the time of the study? | N/A for the story completion task is survey based |  |
| 4. Gender | | Was the researcher male or female? | N/A for the story completion task is survey based |  |
| 5. Experience and training | | What experience or training did the researcher have? | N/A for the story completion task is survey based |  |
| **Relationship with participants** | | | |  |
| 6. Relationship established | | Was a relationship established prior to study commencement? | N/A for the story completion task is survey based |  |
| 7. Participant knowledge of the interviewer | | What did the participants know about the researcher? e.g. personal goals, reasons for doing the research? | P. 8 |  |
| 8. Interviewer characteristics | | What characteristics were reported about the interviewer/facilitator? e.g. Bias, assumptions, reasons and interests in the research topic | P. 8 |  |
| **Domain 2: Study design** | | |  |  |
| **Theoretical framework** | | |  |  |
| 9. Methodological orientation and Theory | What methodological orientation was stated to underpin the study? e.g. grounded theory, discourse analysis, ethnography, phenomenology, content analysis | P. 10 |  |  |
| **Participant selection** | | |  |  |
| 10. Sampling | How were participants selected? e.g., purposive, convenience, consecutive, snowball | P. 9 |  |  |
| 11. Method of approach | How were participants approached? e.g., face-to-face, telephone, mail, email | P. 9 |  |  |
| 12. Sample size | How many participants were in the study? | P. 9 |  |  |
| 13. Non-participation Setting | How many people refused to participate or dropped out? Reasons? | P. 12 |  |  |
| 14. Setting of data collection | Where was the data collected? e.g., home, clinic, workplace | P. 8 |  |  |
| 15. Presence of nonparticipants | Was anyone else present besides the participants and researchers? | N/A for the story completion task is survey based |  |  |
| 16. Description of sample | What are the important characteristics of the sample? e.g. demographic data, date | P. 10 |  |  |
| **Data collection** | | |  | No |
| 17. Interview guide | Were questions, prompts, and guides provided by the authors? Was it pilot tested? | P. 8 |  |  |
| 18. Repeat interviews | Were repeat interviews carried out? If yes, how many? | N/A |  |  |
| 19. Audio/visual recording | Did the research use audio or visual recording to collect the data? | N/A for the story completion task is survey based |  |  |
| 20. Field notes | Were field notes made during and/or after the interview or focus group? | N/A for the story completion task is survey based |  |  |
| 21. Duration | What was the duration of the interviews or focus group? | P. 8 |  |  |
| 22. Data saturation | Was data saturation discussed? | P. 37 |  |  |
| 23. Transcripts returned | Were transcripts returned to participants for comment and/or correction? | N/A for the story completion task is survey based |  |  |
| **Domain 3: analysis and findings** | | |  |  |
| **Data analysis** | | |  |  |
| 24. Number of data coders | How many data coders coded the data? | P. 10 |  |  |
| 25. Description of the coding tree | Did the authors provide a description of the coding tree? | N/A for discourse analysis was adopted |  |  |
| 26. Derivation of themes | Were themes identified in advance or derived from the data? | P. 11 |  |  |
| 27. Software | What software, if applicable, was used to manage the data? | P. 10 |  |  |
| 28. Participant checking | Did participants provide feedback on the findings? | P. 11 |  |  |
| **Reporting** | | |  |  |
| 29. Quotations presented | Were participant quotations presented to illustrate the themes/findings? Was each quotation identified? e.g., participant number | P. 13-29 |  |  |
| 30. Data and findings consistent | Was there consistency between the data presented and the findings? | P. 12-31 |  |  |
| 31. Clarity of major themes | Were major themes clearly presented in the findings? | P. 12-31 |  |  |
| 32. Clarity of minor themes | Is there a description of diverse cases or a discussion of minor themes? | P. 14 |  |  |

**Supplementary Material 2 - Story Stems and Instructions**

***The instructions to participants are provided before the story stem:***

*"You will be provided with a short story that is not yet complete, but you will be asked 3 questions to finish the story. Feel free to be as creative or as simple as you would like with your response! You can write as little or as much as you want, but please try to spend about 10 minutes writing so that we generate some detailed stories"*

***The participants are then randomly assigned to one of 3 story stems: with the three prompts to finish the story:***

*[Story Stem 1]*

*The Palliative Care Committee has recently been established at Acme Hospice. The first meeting was a big success, with representatives from staff on the wards, the affiliated university, the community hospice, and the health administration. One of the Palliative Care Specialist Nurses, Drew, couldn't help but notice that there were no patient or carer representatives at the meeting. They decided that at the next meeting (held next month) they should raise this lack of engagement with the committee.*

*1. Please write about the run-up to the meeting and how Drew prepared for it...*

*2. Please write about the meeting and how the other members responded to Drew's concerns about a lack of patient or carer representatives on the committee...*

*3. Please write about what happened next... (feel free to write as much as you like about any of the characters, and as far into the future as you choose).*

*[Story Stem 2]*

*The Palliative Care Committee has recently been established at Acme Hospice. The first meeting was a big success, with representatives from staff on the wards, the affiliated university, the community hospice, and the health administration. Drew is the committee carer representative - having cared for their partner who died at the hospital a few months ago. Some of the committee members seemed to think that Drew's experiences were not representative of other carers at the hospital. Drew wants to discuss this concern at the next meeting.*

*1. Please write about the run-up to the meeting and how Drew prepared for it.*

*2. Please write about the meeting and how the other members responded to Drew's concerns about a lack of understanding of a representative role on the committee...*

*3. Please write about what happened next... (feel free to write as much as you like about any of the characters, and as far into the future as you choose)*

*[Story Stem 3]*

*The Palliative Care Committee has recently been established at Acme Hospice. The first meeting was a big success, with representatives from staff on the wards, the affiliated university, the community hospice, and the health administration team. Drew works in a quality improvement role and noticed that discussions about informal carer representation on the committee were being led by health professionals rather than carers. Drew wants to suggest at the next meeting that consumers and carers be the ones who decide about representation on the committee.*

*1. Please write about the run-up to the meeting and how Drew prepared for it.*

*2. Please write about the meeting and how the other members responded to Drew's concerns about a lack of patient or carer decision-making within the committee...*

*3. Please write about what happened next... (feel free to write as much as you like about any of the characters, and as far into the future as you choose*

***After the completion of the story, participants were allowed to identify as either a health care provider, an administrator in policy or a consumer of palliative care.***
